# Lectures on Surgical Pathology

**Published:** 1860-01

**Authors:** 


### BOOK AND PAMPHLET NOTICES.

LECTURES ON SURGICAL PATHOLOGY. By James Paget, F. R. S. Second American Edition. Published by Lindsay & Blakiston, Philadelphia. 1860.

This work is a reprint of the previous edition, without material modification, but as it is not yet in every library where it should be, a brief notice of its contents may not be out of place. These lectures were originally delivered at the Royal College of Surgeons, England, and have been enlarged for the press.

The first fifty pages are devoted to a consideration of the nature, purpose, and conditions of nutrition. The positions of the author are illustrated by a variety of cases of pathological nutrition, both in men and animals. After this there is a full discussion of the subjects of hypertrophy, atrophy, the reproduction of lost parts, and the repair of injuries. The principles and processes of these several conditions are richly illustrated by cases from human and comparative pathology. The subject of the repair of bones and tendons is treated with great interest.

Inflammation, with its various products and consequences, next follows, with a pretty full consideration. Morbid growths are next in the order, including the innocent and malignant tumors, and tubercles. These subjects are fully illustrated with engravings, and the cuts showing the microscopic appearances of malignant growths are peculiarly valuable.

Some of the conclusions in this work are not true, others are doubtful, but in the main it is the best and most complete treatise on surgical pathology extant.

